# NEWS

**DOI:** 10.7189/jogh.03.020201

**Published:** 2013-12

## AFRICA

• The African Development Bank published a review of health within Africa over the past 50 years, looking at health patterns and trends, and the challenges of the next 50 years. Fifty years marks the independence of many African countries from colonial rule. It highlights the improvement in life expectancy (from 38 years in 1958 to 56 in 2012), and successes in improving maternal, infant and under–five child mortality rates. In the future, Africa faces the burden of growing non–communicable diseases (eg, cancer, type 2 diabetes), whilst still grappling with high incidences of communicable diseases (eg, malaria, and HIV/AIDS). Moreover, it faces shortages of health professionals and unequal access to health care. Climate change may pose severe threats to health, as changes in temperature, rainfall and humidity could increase vector–borne infectious diseases. (*AfDB*, 31 Mar 2013)

• At the recent World Health Assembly, Nigeria announced a 67% fall in Wild Polio Cases (WPV). It also reported that no type 3 polio virus (WPV3) has been discovered since November 2012, circulating genotypes have collapsed from fourteen to two, and that North West Nigeria is no longer an epicentre of polio. To reach more children, including these in areas where states of emergency had been declared, Nigeria called for more availability of vaccinations that are simple to administer and store. Concerns were raised about the growing cost of child vaccinations, and lack of global control over prices. (*The Guardian*, 23 May 2013)

• The future well-being of millions of African people may rest in the unlikely hands of a vegan hippy scientist. Mr Howard–Yana Shapiro, the agriculture director of the US$ 36 billion US confectionery corporation Mars, who sequenced and published the complete genome of the cacao tree in 2010, now plans to map and then give away the genetic data of 100 traditional crops including yam and cassava. These “orphan crops” are ignored by scientists, seed companies and governments – who all rather focus on maize, rice and soya – but are a staple for up to 250 million smallholder African farmers who depend on them for food security, nutrition and income. Shapiro describes the huge potential to develop more resilient and higher–yielding varieties of most orphan crops by combining traditional plant breeding methods with new biotech tools. “The genetic information will be put on the web and offered free to plant breeders, seed companies and farmers on condition it is not patented. A new African plant–breeding academy will also be set up in Nairobi, Kenya.” It is a gift to start an initiative to boost African farming. (*The Guardian*, 2 Jun 2013)

• The UN warned of a severe outbreak of polio in Somalia, with at least 105 cases of polio have been recorded in Somalia this year – almost half the number of cases around the world in 2012. Somalia was declared polio–free in 2007, and about four million people have been vaccinated. Most of the Somali cases are in areas controlled by militants. The outbreak in Somalia has spread to neighbouring Kenya, where 500 000 Somalis have fled. (*Philadelphia Inquirer*, 17 Aug 2013)

• UN AIDS congratulated the President of Uganda, Yoweri Museveni and the First Lady Janet Kaguta Museveni for their renewed commitment to the Ugandan HIV response. To encourage more Ugandan citizens to know their HIV status, President Museveni took a public HIV test at a health centre in Kampala. At the event, he urged all Ugandans to know their HIV status and to access testing and counselling. He stressed that anyone living with HIV would receive a package of care, treatment and support. He also called on everyone to avoid risky sexual behaviour. The campaign aims to reach 15 million people by the end of 2014. In Uganda, 577 000 people living with HIV are accessing life–saving treatment, and part of the campaign will ensure than an additional 240 000 people will receive treatment by the end of 2014. (*UN AIDS*, 8 Nov 2013)

## ASIA

• A simple invention by Dr Mohammad Abdul Quaiyum at the International Centre for Diarrheal Disease Research in Bangladesh helps to identify life–threatening haemorrhaging in women who have just given birth. He has invented a small birthing mat, which is designed to absorb 400 ml of blood during labour. If a woman loses more, the mat gets saturated, indicating that the woman is haemorrhaging and needs urgent medical help. It was tested by a charity that distributes birthing kits to pregnant women. It was added to their existing kits, with a drug to control haemorrhages. 77 000 kits were distributed, and Dr Quaiyum’s follow–up survey found that 97% said that the mat was beneficial, and that 37 fewer–than–expected new mothers had died – achieved at a cost of 50 cents per mat. (*The Economist*, 18 May 2013)

• Japanese researchers have developed a vaccine that cuts the risk of malaria by 72%. WHO estimates that malaria causes 660 000 deaths per year, mostly children in Africa, where it kills one child each minute. The new vaccine, BK–SE36 targets the most dangerous species of the malaria parasite, *Plasmodium falciparum*, at its blood parasite stage. It underwent initial trials in Japanese adults and has now been tested in a malaria endemic area in Northern Uganda. Its safety and immunogenicity has been assessed, and evidence shows that it will cut malaria infections by 72%. Additional data for safety, tolerability and adverse reactions will be collected in larger trials in younger children. (*PLoS ONE*, 28 May 2013)

• Bangladesh is now one of Asia’s fastest–growing economies, and out–ranks India on some measures of social welfare, such as child mortality. However, its growth potential and ability to lift more of its people out of poverty is hampered by endemic problems in its political system, such as corruption, and the harassment and prosecution of Opposition politicians. Bangladesh’s problems are thrown into relief by the recent Rana Plaza disaster, where 1100 people were killed and 2500 injured by a collapsed factory. The building was deemed unfit, and workers were sent home – but then ordered back to work the day before it collapsed. Culpability has been pinned on the government for not enforcing existing legislation to prevent such tragedies. This lack of enforcement, and related political unrest, is deterring other investors, to the detriment of Bangladesh and its people. (*Financial Times*, 5 Aug 2013)

• WHO and the Damascus government confirmed the first outbreak of polio in war–torn Syria for 15 years, in the eastern province of Deir al–Zour. WHO reported 10 confirmed cases, and another 12 being investigated, all affecting babies and toddlers. Prior to the outbreak of war in 2011, 95% of Syria’s children were immunised. Since then, an estimated 500 000 children are unvaccinated, leaving them at risk of contracting this incurable disease, whose potential complications include paralysis. The city of Deir al–Zour remains in partial control by forces loyal to President Bashar al–Assad while the opposition holds the surrounding countryside. Given the highly dangerous situation, both government and rebel fighters were urged to respect “vaccination ceasefires” to permit access to the hundreds of thousands of children threatened by an outbreak of polio. It is hoped this news will spur international attempts to secure safe passage for humanitarian relief workers. (*The Guardian*, 29 Oct 2013)

• Typhoon Haiyan struck the Philippines in November 2013, causing death and destruction on a massive scale. Homes and crops that survived the first onslaught were left at the mercy of ensuing flooding and landslides. The city of Tacloban felt the full force of the storm. Mr Manuel Roxas, the interior secretary, likened the scale of destruction to a tsunami. Early relief efforts were rendered almost useless as water, electricity and telecommunications were cut off, roads were blocked and bridges swept away. The final death toll may reach 10 000 people, with hundreds of thousands left homeless. Looking ahead, the Philippines disaster risk–reduction and management systems must be overhauled, as they could not cope with Typhoon Haiyan, a disaster on an extraordinary scale. (*The Economist*, 11 Nov 2013)

## AUSTRALIA AND WESTERN PACIFIC

• An Australian proposal to allow childcare centres and schools to ban unvaccinated children has sparked controversy. As a signatory to the UN Convention of the Rights of the Child, Australia agreed that “the best interests of the child shall be a primary consideration.” The Convention states that every child has the right to “the highest attainable standard of health,” and immunisation saves an estimated 2–3 million lives each year, and benefits massively outweigh risks. In recognition, the UN states that all children should be immunised. But banning non–vaccinated children from schools and childcare risks violating Article 28 of the Convention, whereby education is compulsory and freely available. This shows that parental education should be used to ensure vaccination, rather than punitive measures which may violate children’s right to an education. (*Science Alert*, 24 May 2013)

• During a 14–hour visit to Australia, Mr Bill Gates raised an extra US$ 75 million from the Prime Minister for his polio campaign. His major focus is eradicating polio and he's down to just three countries with 300 cases, and gives himself a good chance of eradicating it. Cervical cancer is his next vaccination focus. He pointed out that the Foundation does not invest in tobacco or weapons companies. He believes that governments should tackle corporate tax minimisation and avoidance through laws, rather than expecting companies to do so voluntarily. He also said that he isn’t planning to freeze or clone himself as a way to cheat death. “Life is great but you have to make room for people who come after you,” he said. (*news.com.au*, 24 May 2013)

• The number of children contracting the most common strains of potentially fatal pneumococcal disease fell by 97% in Australia since universal vaccination began in 2005. Unvaccinated adults also benefit from the widespread immunization of infants. It mainly affects children under two and the elderly, and those who develop meningitis risk brain damage or death. Since the introduction of the universal vaccination for under–twos in 2005, severe infection caused by the seven most common strains of pneumococci had plunged from 60.9 to 2.1 per 100 000. More work is needed to increase uptake of the vaccine amongst Australia’s indigenous people and other groups with certain risk factors. (*The Conversation*, 27 May 2013)

• Australia has become the first country to offer the human papilloma virus (HPV) vaccine to 12-year–old boys. It has been available since 2007 and is administered to girls to prevent HPV infections and associated cervical cancer. Australia has a higher mortality rate in men from HPV–associated cancers (mainly oral cancers), compared to women. Worldwide, an estimated 600 000 people develop an HPV–related form of cancer annually, with a 50% mortality rate. The current vaccination rate needs to be maintained and improved, and distributed globally, as the proportion of herd immunity needed to stop the spread of infection is not yet understood. On–going trials aim to develop further immunotherapies, protecting both men and women. (*The Guardian*, 4 June 2013)

• An Australian bill to guarantee public access to patented drugs, vaccines and genetic tests, and to allow Australia to export cheaper and generic versions of vital drugs passed its first parliamentary hurdle. The bill attracted some criticism for being inconsistent with Australia’s free–trade agreement with the USA, and for contradicting a World Trade Organisation (WTO) agreement that drugs can only be exported to WTO–member countries. In reply, Australia’s Parliamentary Secretary for Innovation, Ms Yvette D’Ath, stated that “arguably, non–WTO members are the countries that need our help the most.” (*Sydney Morning Post*, 26 June 2013)

## CHINA

• Chinese life expectancy at birth increased from 69.3 years to 75.7 years between 1990 and 2010, largely due to decreasing child mortality and lower rates of tuberculosis and lower respiratory infections. Child mortality fell by 6% per annum, mainly due to massive reductions in childhood respiratory infections and diarrhoea. This gives China the lowest rate of premature mortality amongst the developing countries, and a rate only marginally higher than the USA. However, this is partially offset by growing concerns over the rise of western–style diseases in China, with increasing non–communicable diseases (eg, dementia), driven by urbanisation and an ageing population, plus lifestyle diseases (eg, type 2 diabetes, lung cancer). There are calls for more aggressive tobacco control to help address these emerging problems. (*Medical News Today*, 8 Jun 2013)

• The rapid pace of growth in the Chinese economy is associated with an exponential increase in China’s aid to developing countries over the last decade. By 2009, China provided US$ 1.4 billion in aid, including at least 49 African countries, making it an emerging strong player in global health. Its development approach introduces qualitative changes to the cost–effectiveness focus of western frameworks, moving towards more solidarity and mutual benefit approaches in this so called ‘south–south’ co–operation. While helping others, China will also need to deal with its own troubling double burden of disease, the large internal market for counterfeit medicines and concerns about transparency and human rights in its policies. The post–2015 development agenda will become a critical period for Chinese involvement in global health. (*The Guardian*, 10 Jun 2013)

• The British pharmaceutical company GSK recently sacked the head of the Research and Development department of its Chinese branch. A Chinese neurologist was dismissed after a study he co–authored and published in *Nature* in 2010 contained misrepresented data. The study aimed to elucidate the role of the protein interleukin–7 in autoimmune diseases. The authors used blood samples from healthy subjects, but reported them as belonging to patients with multiple sclerosis, thus skewing the results. Afterwards, another author resigned and three were placed on administrative leave. Trials of an experimental multiple sclerosis drug informed by the study’s results have been suspended as a safety measure. (*Reuters,* 11 June 2013)

• China’s Health Ministry announced plans to monitor and evaluate the long–term impact of chronic air pollution on human health. It will gather data on particulate matter with a diameter of 2.5 μm (PM2.5) in different locations. It will uncover linkages between air pollution and health, initially focusing on cities where pollution is most prevalent. It will analyse PM2.5 data, weather information and local disease and death. It is estimated that poor air quality can shorten peoples’ lives by 5.5 years, and air pollution and quality are of growing concern to China’s increasingly urbanised population. Recently, PM2.5 levels reached 1000 in some parts of Harbin, a city of 11 million people, and brought the city to a virtual standstill. A level above 300 is hazardous, and WHO recommends a limit of 20. (*Reuters*, 28 Oct 2013)

• In November 2013, Mr Xi Jinping, China’s party chief and state president, convened the five–yearly plenary meeting of the 11th central committee. It is hoped that Mr Xi will use the meeting to drive changes in state–owned enterprises and in the countryside, where farmers still lack clear rights to their land – in stark contrast to city–dwellers’ property rights. Reforms would boost China’s trajectory from an investment–heavy economy, supported by cheap labour, to a mature model based on consumption and high wages. (*The Economist*, 2 Nov 2013)

## EUROPE

• Bernia Hamidović was a three–month–old baby who died after her lack of an ID number prevented her treatment for a tracheoesophageal fistula. Her parents tried to take her across the border to Belgrade for treatment, after treatment in Sarajevo failed. She did not have a passport or medical card, as the parliamentary failures to define Bosnian regions (determining ID numbers) means that children born after February 2013 cannot be registered. The debate is sparked by a push to recognise the geographical split between the Serb majority region and Croat–Bosniak majority region. Although Bernia was allowed to travel to Belgrade, she was refused treatment as funding was denied. She died from infection before Serbian doctors could carry out treatment. (*Fox News*, 17 Jun 2013)

• Across Europe, over 50% of people are overweight or obese and non–communicable diseases cause 77% of the disease burden. At the WHO's European Ministerial Conference of Health 2020, ministers of health pledged to reduce obesity and promote health. They pledged to improve monitoring of the impact of the problem, and to more effectively address the root causes of obesity. Possible actions include: restricting the marketing of junk food to children; ensuring that the food industry does more to tackle health problems; more intensive monitoring of key issues; and supporting healthier food choices. (*WHO Europe,* 5 July 2013)

• The European Respiratory Society evaluated the European disease burden from respiratory conditions, using data from WHO and the European Centre for Disease Prevention and Control. It found that deaths from diseases such as lower respiratory tract infections, lung cancers, tuberculosis and Chronic Obstructive Pulmonary Disease account for 10% of deaths across Europe. Mortality from these diseases is highest in the UK and Ireland, whereas Finland and Sweden have the lowest rates. Smoking is the largest preventable cause of these illnesses. “Both the prevention and treatment of lung diseases will need to be improved if their impact on longevity, quality of life of individuals and economic burden on society are to be reduced in Europe and worldwide,” said the president of the European Respiratory Society, Prof Francesco Blasi. (*BBC News*, 6 Sept 2013)

• The European Parliament rejected proposals to regulate e–cigarettes as medicine, which could have restricted their availability and their up–take by smokers. E–cigarettes contain a liquid nicotine solution that is inhaled without burning tobacco and its associated carcinogenic side–effects. Some analysts expect e–cigarettes to supplant traditional cigarettes. They will still be subject to the same marketing restrictions as tobacco. At the same time, the Parliament voted to increase the size of warning labels on cigarette packets, and approved a ban on menthol and other flavoured cigarettes designed to appeal to youngsters. (*Financial Times*, 8 Oct 2013)

• A UK Government report shows that UK child mortality rates are amongst the worst in Europe, worsening over the past 15 years. This translates into an additional 2000 children dying each year in the UK, compared to other Western European countries. Wide variations in care and in the management of common conditions were highlighted. Underlying problems may be exacerbated by the UK’s high rates of relative poverty, with its implications for health outcomes. As well as moral and social imperatives, there are economic arguments for investing in children’s health, as poor health in childhood can store up future problems. (*The Guardian*, 30 Oct 2013)

## INDIA

• India’s leading drug company, Sun Pharmaceutical Industries, is in talks with the Swedish drug–manufacturer Meda AB to buy a controlling stake for US$ 5–6 billion, primarily to boost its generics business in developed markets. With the same focus as Sun Pharma, Meda is involved in speciality products, over–the–counter drugs and branded generics, and had sales of US$ 1.98 billion in 2012. Sun Pharma has made several acquisitions in recent years, but this would be its biggest so far. Last year, it bought US–based Dusa Pharmaceuticals Inc. for US$ 230 million, and URL Pharma from Japan's Takeda Pharmaceutical Co for an undisclosed amount. Buying Meda gives Sun Pharma access to Dymista, an allergy medicine with high market potential, which received US approval in 2012. (*Reuters*, 31 May 2013)

• Research published in *The Lancet* suggests that 45% of deaths in children under five are caused by malnutrition. A review of maternal and child under–nutrition and obesity in low– and middle–income countries, including a progress review for nutrition programmes, was led by Prof. Robert Black from Johns Hopkins Bloomberg School of Public Health in Baltimore, USA. Despite progress, 165 million children are affected by stunting and 50 million by wasting (low weight for height) in 2011, with India being the largest single contributor. An estimated 900 000 lives could be saved in 34 countries if 10 proven nutritional interventions were scaled–up to 90% of the world. The researchers warn that countries will not break out of poverty unless nutrition becomes a global priority. “If maternal and child nutrition can be optimised, the benefits will accrue and extend over generations, which is why we must work together now to seize this opportunity,” said Dr Richard Horton, Editor–in–Chief of *The Lancet*. (*The Lancet*, 3 Aug 2013)

• India’s cheap food scheme, which aims to reach 800 million people, was enshrined in law in August 2013. Its strengths include ensuring children have a daily hot lunch and promoting better nutritional advice and health–care for under–sixes. Critics say that it does not address the huge problem of food storage and waste in India, and is not effectively targeted at the 20 million people who most need help. More emphasis is needed on better nutrition, as undernourishment is common even where food supplies are adequate. Roughly half of children under five are undernourished, and 60 million are stunted, and face brain damage, reduced capacity to learn and higher mortality. There are calls for more spending on public health and better sanitation, as infections from polluted water affect peoples’ ability to absorb nutrients. (*The Economist*, 24 Aug 2013)

• Pathfinder International, which aims to expand access to quality sexual and reproductive health care, announced a new partnership with the state government in Haryana, India. Pathfinder will provide technical support and guidance, drawing on its history of working to improve sexual and reproductive health in India. A large part will focus on changing traditional patterns of behaviour, such as delaying ages of marriage and first pregnancy, and spacing births. The project will sensitize men and religious leaders in the community to the importance of equality and the healthy timing and spacing of pregnancy. Pathfinder will provide support for capacity building and supportive supervision of frontline health workers to help streamline the contraceptive supply chain system to better serve communities. (*Pathfinder*, 30 Sept 2013)

• The annual Global Hand–washing Day on 15 October was organised by the Global Public–Private Partnership for Hand–washing with Soap. It supports the universal promotion and practice of proper hand–washing with soap at critical times. This stops the transmission of disease agents and can significantly reduce diarrhoea and respiratory infections, and may impact on skin and eye infections. It is an extremely effective and inexpensive way to prevent infections responsible for millions of child deaths, with India being the country where the largest number of related child deaths occurs. Turning the simple steps of hand–washing with soap before eating and after using the toilet into ingrained habits could help towards meeting the Millennium Development Goal of reducing deaths amongst children under the age of by two–thirds by 2015. (*Partnership for Handwashing,* 15 Oct 2013)

## THE AMERICAS

• In the wake of the devastating earthquake in 2010 which killed over 200 000 people and displaced over one million people, there has been the worst cholera outbreak in recent history, and has claimed 8000 lives to date. The rapid and focused response has averted more casualties and the case–fatality rate has dropped below the WHO standard of 1%. However, Haiti will probably have on–going problems with cholera infections. It is vital that these are addressed by investing in Haiti’s water and sanitation infrastructures. To achieve this, the Pan–American Health Organisation, the US Centers for Diseases Control and Prevention and UNICEF have joined with the governments of Haiti and the Dominican Republic to develop plans for cholera elimination. The US$ 2.2 billion programme will focus on improving water, sanitation and hygiene conditions that are the main vector for cholera infections. (*PAHO*, 9 Oct 2013)

• A letter to President Obama calls for an end to the US embargo against Cuba. It arose from a critical care conference held in Havana, which focused on the embargo’s devastating effects on Cuban health. The embargo prohibits US companies from selling medical supplies to Cuba, and prevents foreign companies from trading with the US if they do business with Cuba. At the conference, Jorge Soberon of the Cuban Health Ministry, reported that this prevented Cuba from accepting US$ 4 million from France to tackle AIDS and tuberculosis, and led to problems in treating children with diseases such as leukaemia and diarrhoea. Doctors from 18 countries, including the USA, attended the conference, and called the embargo a “humanitarian catastrophe.” (*BMJ*, 29 Oct 2013)

• Three projects were honoured at the annual Malaria Day as “malaria champions of the Americas”, which integrate malaria interventions with solutions for other health problems. In the Americas, nearly 106 million people live in areas at high risk of malaria, although the number of cases fell by 60% from 2000 to 2012, and the number of deaths fell by 70%. The main award went to the Colombia Malaria Project, whose achievements include creating sustainable local capacity through training local health workers in malaria prevention and control, and improving the well–being of indigenous communities. The National Centre for Control of Tropical Diseases in the Dominican Republic was recognized for its innovative use of technology in addressing each malaria case individually, and collaborations with other agencies. The Secretariat of Health of the State of Acre, Brazil was recognised for leadership in reducing malaria, and its efforts to address other health problems. (*PAHO*, 7 Nov 2013)

• According to the recent Pan–American Health Organisation (PAHO) recent report on human resources in health, about 70% of countries in the Americas have sufficient (or even more) doctors, nurses and midwives to provide basic health care services for their populations, with ratios at or above WHO/PAHO recommendations. However, many countries face challenges in the distribution, training and migration of workers. Healthcare workers are often concentrated in urban areas at the expense of inaccessible and sparsely populated areas. Some countries face high rates of outward migration of health care workers, leading to shortages, so improving workers’ employment is important. Looking to the future, training must be aligned with changing health needs, and focus on health care access, equity and quality. (*PAHO*, 11 Nov 2013)

• As OECD research shows that young people in the USA’s workforce have the lowest levels of maths skills in the developed world, a new experiment is under way in raising the educational attainment of the poorest children in the USA. Poor children’s attainment lags behind their wealthier peers at the start of school, and the gap persists throughout the child’s education. Prof Greg Duncan, a US economist, will take a randomized group of low–income single mothers, and give each US$ 4000 for the first three years of the children’s lives. A control group will get smaller amounts. The research aims to establish if there is a direct link between poverty reduction and cognitive development, and if children’s school performances improve as a result. Improving educational performance is the key to boosting the US’s low social mobility rate. (*BBC News*, 13 Nov 2013)

